# Evaluation of a fast kV switching dual energy CT in deriving relative stopping power using tissue equivalent phantoms of various sizes

**DOI:** 10.1002/mp.70288

**Published:** 2026-01-24

**Authors:** Hazel Wang, Yanling Qu, Yang Li, Paul Deak, Mark Pankuch

**Affiliations:** ^1^ Northwestern Medicine Medical Physics Warrenville USA; ^2^ GE Healthcare CT Engineering Beijing China; ^3^ GE Healthcare Research Glattbrugg Switzerland

**Keywords:** dual energy CT, proton therapy planning, relative stopping power

## Abstract

**Background:**

Dual energy CT (DECT) has been demonstrated to improve relative stopping power (RSP) estimation with knowledge of the effective atomic number ($Z_{\mathrm{eff}}$) and relative electron density (RED). A fast kV switching DECT has been developed and it is necessary to quantify the accuracy of RSP prior to clinical use.

**Purpose:**

This study evaluates the accuracy of a fast kV switching DECT to calculate $Z_{\mathrm{eff}}$, RED, and RSP in tissue‐equivalent phantoms of various sizes.

**Methods:**

Five different phantoms containing tissue‐mimicking inserts were scanned using the Body Scanning Field of View (SFOV) of a fast kV switching DECT. Head phantoms were rescanned with the Head SFOV. Data from the DECT was used to obtain voxel‐matched $Z_{\mathrm{eff}}$ and RED volumetric images. From these data, a volumetric image of RSP was calculated for each voxel in the volume. Derived values were compared to their corresponding reference values.

**Results:**

The largest differences were seen in the low‐density inserts. The mean absolute percent error (MAPE) of $Z_{\mathrm{eff}}$ and RED was 2.02% ± 2.48% and 1.29% ± 1.86% respectively. RSP MAPE was 1.13% ± 1.17% with the largest difference in sinus material at 6.3%. When comparing DECT‐derived values obtained using Body versus Head SFOV, a paired *t*‐test showed no significant difference (*p* = 0.61).

**Conclusions:**

Ultra‐fast kV switching DECT can be used to predict RSP in phantom with an accuracy that is non‐inferior to the range uncertainties commonly used in the clinical environment and has the potential to be used for proton therapy planning.

## 1 INTRODUCTION

Proton treatment plans rely on the accurate prediction of proton stopping power relative to water (RSP) in order to calculate the proton range necessary to adequately cover a target volume. Without the proper range determination, the proton beam might under‐range and consequently under‐dose the targeted area, or the proton beam might over‐range and unnecessarily over‐dose surrounding downstream tissues. To mitigate the potential of under‐dosing the target areas as a result of uncertainties in the proton range calculation, safety margins on the order of 3.5% of the incident proton range are applied both upstream and downstream of the proton beam.^1^, ^2^, ^3^ This prioritization of target coverage comes at the cost of additional dose to nearby healthy tissues making it highly desirable to minimize proton range calculation uncertainties and the corresponding extended margins.

A contributing factor to the uncertainty of calculating the proton range is the conversion of the CT number Hounsfield Unit (HU) from the treatment planning CT to RSP.^2^, ^4^ The treatment planning CT that is conventionally used is based on single‐energy (SECT) acquisition. The HU of a SECT is derived using the linear attenuation coefficient of a material and is a function of its effective atomic number, the mass density, and the imaging spectrum of the photon source. HU consistency can also be affected by scanning conditions such as scanning parameters, the presence of high‐density materials, and object size.^1^, ^2^, ^3^, ^4^

Dual energy CT (DECT) is an image acquisition method that utilizes both a low energy and a high energy while acquiring the image dataset. There are a variety of configurations of source and detector DECT, which can be referenced in the literature.^5^, ^6^ A number of studies have shown that DECT can improve the accuracy in the RSP calculation due to its ability to derive relative electron density (RED) and effective atomic number $(Z_{\mathrm{eff}}$) maps.^7^, ^8^, ^9^, ^10^, ^11^, ^12^ By extracting these values in a voxel‐wise matrix, proton RSP can be calculated for each voxel of the image set using the Bethe–Bloch equation.

(1) $$\mathrm{RSP}=\frac{\rho_{e,m}\left[\ln\left(\frac{2m_{e}c^{2}\beta^{2}}{\left(1-\beta^{2}\right)}\right)-\beta^{2}-\ln I_{m}\right]}{\rho_{e,w}\left[\ln\left(\frac{2m_{e}c^{2}\beta^{2}}{\left(1-\beta^{2}\right)}\right)-\beta^{2}-\mathrm{In}\;I_{w}\right]}$$

where $m_{e}$ is the rest mass of the electron, c is the speed of light in vacuum, $\rho_{e,m}/\rho_{e,w}$ is RED, $I_{m}$ is the mean excitation energy of the material, $I_{w}$ for water is 75.3 eV, and β is speed of protons relative to *c*. The *I*‐value of the material can be determined by the Bragg‐Additivity Rule

(2) $$\ln\;I_{m}=\frac{\sum_{j=1}^{J}\frac{w_{j}Z_{j}}{A_{j}}\ln I_{j}}{\sum_{j=1}^{J}\frac{w_{j}Z_{j}}{A_{j}}}$$

where $w_{j}$ is the mass weight, $Z_{j}$ is the effective atomic number, and $A_{j}$ is the mass number. In clinical practice, $I_{m}$ is empirically correlated with $Z_{\mathrm{eff}}$. Consequently, once $Z_{\mathrm{eff}}$ and RED are determined by a dual energy scan, the RSP can be calculated using Equations 1 and 2, which allows for a more accurate, direct calculation of the RSP. This more accurate calculation of RSP provides additional confidence in the distal, high dose gradients of proton therapy and reduces the range uncertainty margins needed in treatment planning.

There have been a number of studies that have utilized tissue equivalent material to evaluate the ability of DECT to accurately derive RSP.^11^, ^13^, ^14^, ^15^ These studies summarized accuracy of their DECT with a single scanning condition and a single phantom size with inserts in a single position. However, this type of evaluation does not entirely describe the impact on the RSP accuracy for clinical situations encountered; such as differences in body size, artifacts caused by high densities, and beam hardening corrections which could alter the measured CT number.^1^, ^16^

In this study, the accuracy of a fast kV switching dual energy CT (DECT) (GE Revolution, GE Medical Systems, Milwaukee, WI, USA) in calculating $Z_{\mathrm{eff}}$, RED, and RSP is evaluated using phantoms of various sizes with a variety of tissue‐equivalent inserts. In an earlier study, this DECT's RSP accuracy was found to have a mean absolute percent error (MAPE) of 1.4% with the largest percent error of −3.9% seen in the LN‐300 plug.^17^ When the Scanning Field of View (SFOV) was changed to a Head bowtie filter, it was found to give significantly different results than when using a standard Body bowtie filter (*p* = 0.01). Improvements have since been made in the reconstruction of RED and $Z_{\mathrm{eff}}$ maps as well as with scanning with the Head bowtie filter. The results of the accuracy with the reconstruction improvements are presented here.

## 2 METHODS

### 2.1 Phantoms

**FIGURE 1:**
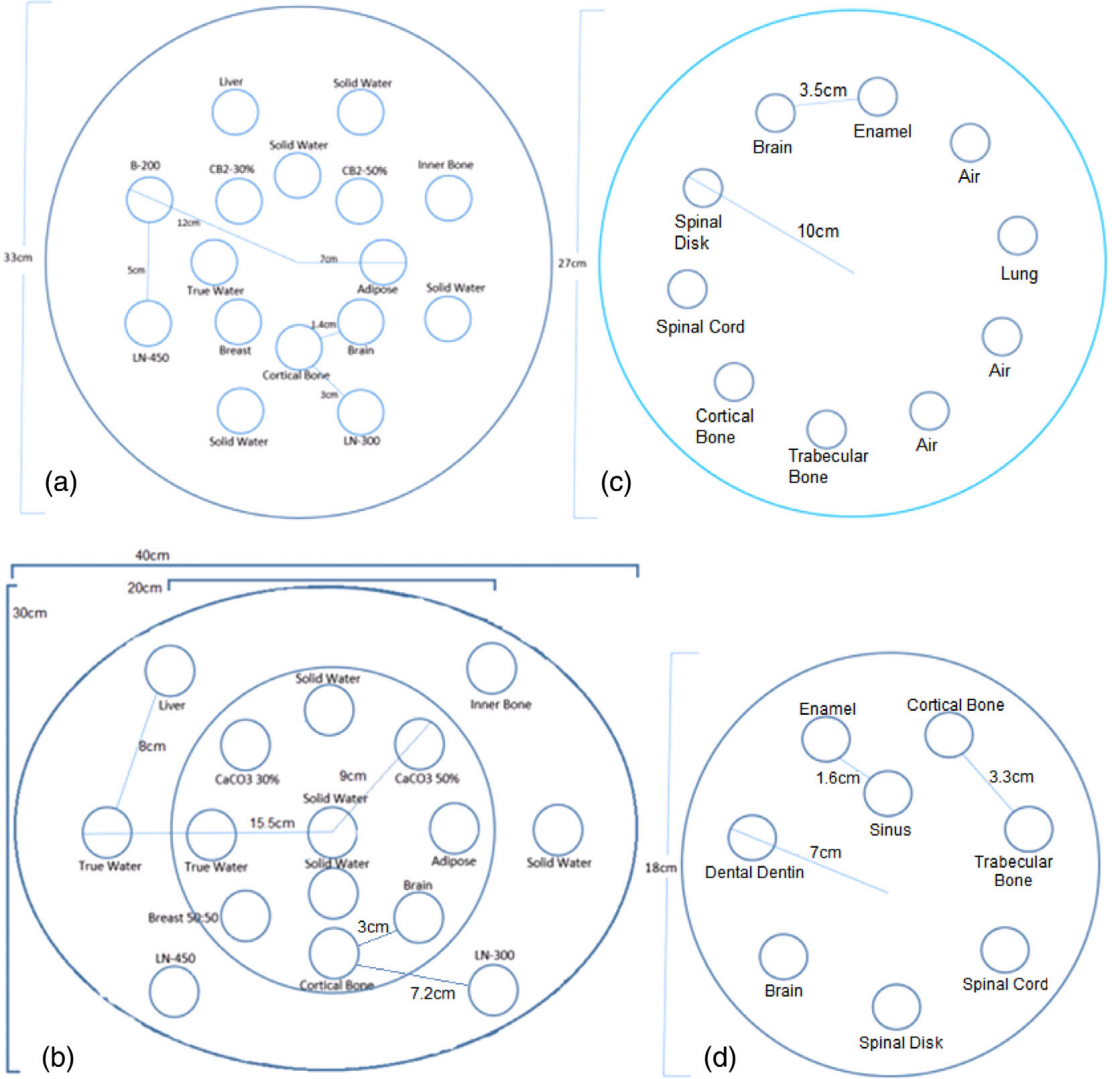
(a) Tissue mimicking phantoms. Model 467 Phantom consisted of an outer and inner ring of inserts. The outer inserts are 12 cm from the center to the edge and spaced 5 cm apart. The inner ring is 7 cm from the center to the outer edge and spaced 1.4 cm apart. The distance from the outer insert to the closest inner insert is 3 cm. (b) Advanced Electron Density phantom consisted of 6 outer inserts on the body section and 10 inserts within the head portion. (c) George Body phantom consisted of a single ring of inserts 10 cm from the center to the outer edge and spaced 3.5 cm apart. (d) George Head phantom consisted of a single ring 7 cm from the center to the outer edge spaced 3.3 cm apart. A sinus insert is located between dental enamel and cortical bone 1.6 cm away from their edge.

Five total phantoms were used in this study: A Tissue Characterization Phantom Model 467 (Gammex RMI, Middleton, WI, USA), the Advanced Electron Density Body Phantom with Head Insert (Sun Nuclear Corporation, Middleton, WI, USA), and two custom, in‐house phantoms, referenced as the George Head and George Body phantoms. The Model 467 is a tissue‐mimicking phantom that is 33 cm in diameter and 5 cm in height. Various tissue‐equivalent inserts ranging from RED 0.93–1.69 were arranged within the solid water phantom according to the manufacturer's recommendations for minimizing artifacts from high density.^18^ The manufacturer also provides the RED of each insert material. Figure 1a shows the schematics of the Model 467 phantom and insert arrangement.

The Advanced Electron Density Phantom is an oblong‐shaped HE CT Solid Water phantom with a 20 cm diameter removable head insert. The body is 40 cm × 30 cm in‐plane, for an equivalent circle diameter of 34.6 cm and 16.5 cm deep. The arrangement of plugs was setup to closely match that of the Model 467 configuration. This can be seen in Figure 1b. The manufacturer also provided the RED of each plug, which ranged from RED 0.94 to 1.78.

Figure 1c,d displays the details of the George Body and Head phantoms, which were constructed from blue wax. The composition of each insert was known and the RED was calculated as described in Section 2.2 (Equation 4). The George Body phantom is 27 cm in diameter with inserts ranging from RED 1.032 to 1.875 and the George Head phantom is 18 cm in diameter with inserts ranging from RED 0.2 to 1.875.

### 2.2 Reference Values

Ground truth reference values were established for $Z_{\mathrm{eff}}$, RED, and RSP. The material composition of each insert provided by the manufacturer can be used to calculate the $Z_{\mathrm{eff}}$ using the Mayneord equation

(3) $$Z_{\mathrm{eff}}={\left[\frac{\sum w_{i}\left(\frac{Zi}{Ai}\right)Zi^{n}}{\sum w_{i}\left(\frac{Zi}{Ai}\right)}\right]}^{1/n}$$

where *w* is weight of the elemental components, *Z* is the atomic number of the element, *A* is the atomic mass of the element, and *n* = 3.21.

A theoretical reference RED can be calculated according to

(4) $$\rho_{e}=\frac{\rho N_{A}\sum w_{i}\left(\frac{Zi}{Ai}\right)}{\rho_{e,w}}$$

where $N_{A}$ is Avogadro's number, ρ is mass density, and $\rho_{e,w}$ is the electron density of water. For the Model 467 and Advanced Electron Density phantoms, the manufacturer supplied a corrected RED for each plug. This correction scales the RED based on a measurement of each rod's physical density compared to the nominal physical density.^18^, ^19^ For this reason, the plugs with the corrected RED were used as the ground truth reference. For the custom‐designed George phantom, there was no supplied RED and Equation 4 was used to calculate the reference RED. Compared to the theoretical value of RED using Equation 4, the manufacturer‐specified RED from Gammex and Sun Nuclear was within ±0.02. Each insert was classified based on its RED value as either air/lung (0.28–0.44), fat (0.93–0.97), muscle (1.02–1.08), water (0.99–1.00), or bone (1.09–1.78).

For RSP, the theoretical Bethe–Bloch equation relies on the $I_{m}$, RED, and $Z_{\mathrm{eff}}$ of the material. Although the phantom manufacturer provides specifications of RED and $Z_{\mathrm{eff}}$, there is still an uncertainty in the manufacturing of the inserts, which results in variability in the final product. Direct measurements of RSP using the clinical proton beam account for the variations within the manufacturing process. Therefore, the ground truth values for RSP were established with direct measurements in order to accurately reflect the actual material as opposed to the theoretical Bethe–Bloch equation, which assumes an ideal material. Direct measurements were performed using transmission measurements with a multilayer ion chamber (MLIC). An MLIC is a stack of ion chamber detectors used to measure proton range. The difference of the measured proton range, defined at the distal D90% of the descending Bragg peak, with and without the plug in the beam's path, provides the water equivalent thickness (WET) of the material. The physical thickness (*t*) of each plug insert, measured with calipers, can be used to determine RSP using:

(5) $$\mathrm{RSP}=\frac{WET}{t}$$

In the George phantoms, the common insert's RSP was previously determined by DeJong et al. using the above MLIC method and Equation 5.^20^, ^21^ The RSP measurement and uncertainty for the Model 467 and Advanced Electron Density inserts were derived using the same methods used by DeJong.

### 2.3 DECT Measurements

The fast kV switching DECT that was used consists of a single source and a single detector that rapidly alternates between 80 and 140 kVp at sub‐millisecond speed for a cycle time of 0.25 ms.^22^, ^23^ All of the phantoms were scanned using the SFOV Large Body. The head phantoms, including the Model 467 phantom, were rescanned using SFOV Head. All phantoms were centered in the bore and helically scanned with a scanning thickness of 2.5 mm using the highest mA setting available to minimize noise. The highest achievable current settings were 480 mA for Body SFOV and 475 mA for Head SFOV. The resulting CTDI_vol_ was 18.85 mGy for the George and Advanced Electron Density phantoms using a rotation time of 0.8 s. The rotation time of 0.6 s was inadvertently set when scanning the Model 467 phantom, which resulted in a CTDI_vol_ of 14.55 mGy. For all Head SFOV scans, the CTDI_vol_ was 82.77 mGy using a 0.8 s rotation time. For all scans, the primary reconstruction used was a standard kernel. Filtered back projection was used for the Body SFOV, while ASIR‐V and 50% level were used for the Head SFOV.

$Z_{\mathrm{eff}}$, RED, and RSP maps were reconstructed with GE software's propriety algorithm using the raw data from the DECT. For all ROI evaluations, an ROI that was at least 60% of the insert was contoured for each plug and the mean value of $Z_{\mathrm{eff}}$, RED, and RSP was reported.

The accuracy of the DECT‐derived values for $Z_{\mathrm{eff}}$, RED, and RSP was quantified against the reference values using percent error. The percent error was compared to the reference value according to

(6) $$\%\mathrm{ERR}\;\frac{=\mathrm{RO}I_{\mathrm{mean}}-X_{\mathrm{ref}}}{X_{\mathrm{ref}}}*100$$

where $X_{\mathrm{ref}}$ is the reference for either the $Z_{\mathrm{eff}}$, RED, or RSP value being investigated.

Since the average of the percent errors may cancel out between a distribution of negative and positive percent errors, the overall accuracy across all inserts was evaluated using the Mean Absolute Percent Error (MAPE). The MAPE is defined as

(7) $$\mathrm{MAPE}\;\frac{=\sum \left|\%\mathrm{ERR}\right|}{n}$$

where *n* is the number of inserts evaluated.

## 3 RESULTS

### 3.1 SFOV Large Body

Table 1 displays each phantom's inserts with the RSP derived from the DECT according to the set SFOV, as well as the reference values used for RED_REF_, $Z_{\mathrm{eff},\mathrm{REF}}$, and RSP_REF_ for each insert. The RSP residual error, *ε*, which is the numerator in Equation 6, and resulting percent error are also provided. The average residual error was −0.002 ± 0.015. All of the residual errors were within 2 S.D. with the exception of the enamel insert and brain insert from Model 467.

**TABLE 1:** Relative stopping power (RSP) dual energy CT (DECT) measurements and insert references.

		References			Head SFOV			Body SFOV		
Phantom	Plugs	RED_REF_	ZEff_REF_	RSP_REF_	RSP_mean_	RSP (ε)	PE (%)	RSP_mean_	RSP (ε)	PE (%)
Model 467	Adipose	0.93	6.14	0.939 ± 0.003	0.938	0.000	**−0.02**	0.935	−0.003	**−0.33**
	B‐200	1.10	10.11	1.108 ± 0.003	1.098	−0.011	**−0.90**	1.097	−0.012	**−1.07**
	Brain	1.04	7.4	1.069 ± 0.003	1.036	−0.034	**−3.15**	1.031	−0.038	**−3.55**
	Breast	0.96	6.82	0.976 ± 0.003	0.972	−0.004	**−0.46**	0.961	−0.015	**−1.55**
	CB2‐30%	1.28	10.78	1.267 ± 0.002	1.255	−0.012	**−0.94**	1.251	−0.017	**−1.31**
	CB2‐50%	1.47	12.40	1.427 ± 0.002	1.429	0.002	**0.14**	1.414	−0.013	**−0.89**
	Cortical bone	1.69	13.53	1.616 ± 0.005	1.630	0.014	**0.89**	1.623	0.008	**0.46**
	Inner bone	1.09	10.11	1.090 ± 0.003	1.083	−0.007	**−0.66**	1.077	−0.013	**−1.17**
	Liver	1.06	7.59	1.080 ± 0.003	1.067	−0.013	**−1.17**	1.061	−0.019	**−1.76**
	LN‐300	0.29	7.61	0.263 ± 0.010	0.282	0.019	**7.07**	0.276	0.013	**4.90**
	LN‐450	0.44	7.58	0.459 ± 0.006	0.463	0.004	**0.85**	0.472	0.013	**2.86**
	Solid Water 1	0.99	7.59	1.005 ± 0.003	0.997	−0.008	**−0.83**	0.991	−0.015	**−1.48**
	Solid Water 2	0.99	7.59	1.005 ± 0.003	0.998	−0.008	**−0.79**	0.996	−0.010	**−0.95**
	Solid Water 3	0.99	7.59	1.005 ± 0.003	0.999	−0.006	**−0.63**	0.992	−0.014	**−1.34**
	Solid Water 4	0.99	7.59	1.005 ± 0.003	1.001	−0.005	**−0.49**	0.989	−0.016	**−1.61**
	True Water	1.00	7.42	1.000 ± 0.000	0.998	−0.002	**−0.19**	0.993	−0.007	**−0.72**
George Body	Brain	1.039	7.48	1.040 ± 0.003	–	–	–	1.044	0.004	**0.36**
	Cortical bone	1.635	13.06	1.555 ± 0.004	–	–	–	1.526	−0.029	**−1.89**
	Enamel	1.875	17.20	1.755 ± 0.004	–	–	–	1.792	0.037	**2.10**
	Spinal cord	1.032	7.47	1.040 ± 0.003	–	–	–	1.042	0.002	**0.21**
	Spinal disk	1.058	7.96	1.070 ± 0.003	–	–	–	1.087	0.017	**1.63**
	Trabecular bone	1.100	9.12	1.100 ± 0.003	–	–	–	1.081	−0.019	**−1.70**
George Head	Brain	1.039	7.48	1.040 ± 0.003	1.045	0.005	**0.46**	1.046	0.006	**0.56**
	Cortical bone	1.635	13.06	1.555 ± 0.004	1.527	−0.028	**−1.78**	1.547	−0.008	**−0.55**
	Dentin	1.555	14.00	1.495 ± 0.004	1.472	−0.024	**−1.57**	1.489	−0.006	**−0.42**
	Enamel	1.875	17.20	1.755 ± 0.004	1.790	0.035	**1.98**	1.812	0.057	**3.26**
	Sinus	0.200	7.42	0.200 ± 0.005	0.225	0.025	**12.40**	0.213	0.013	**6.30**
	Spinal cord	1.032	7.47	1.040 ± 0.003	1.043	0.003	**0.33**	1.044	0.004	**0.42**
	Spinal disk	1.058	7.96	1.070 ± 0.003	1.087	0.017	**1.57**	1.089	0.019	**1.74**
	Trabecular bone	1.100	9.12	1.100 ± 0.003	1.081	−0.019	**−1.76**	1.088	−0.012	**−1.12**
Advanced Electron Density Body	Adipose	0.94	6.61	0.960 ± 0.002	–	–	–	0.956	−0.004	**−0.40**
	Brain	1.02	7.63	1.028 ± 0.002	–	–	–	1.027	−0.001	**−0.11**
	Breast	0.97	6.92	0.981 ± 0.002	–	–	–	0.977	−0.004	**−0.40**
	CaCO3 30%	1.27	10.78	1.261 ± 0.002	–	–	–	1.260	0.000	**−0.03**
	CaCO3 50%	1.46	12.40	1.451 ± 0.002	–	–	–	1.437	−0.013	**−0.92**
	Cortical bone	1.78	13.63	1.699 ± 0.002	–	–	–	1.721	0.022	**1.28**
	Inner bone	1.16	10.39	1.143 ± 0.002	–	–	–	1.148	0.005	**0.45**
	Liver	1.05	7.61	1.059 ± 0.002	–	–	–	1.054	−0.004	**−0.43**
	LN‐300	0.28	7.61	0.289 ± 0.001	–	–	–	0.292	0.003	**1.11**
	LN‐450	0.44	7.58	0.477 ± 0.001	–	–	–	0.487	0.010	**2.14**
	Solid Water 1	1.00	7.45	1.002 ± 0.002	–	–	–	0.997	−0.005	**−0.48**
	Solid Water 2	1.00	7.45	1.002 ± 0.002	–	–	–	0.996	−0.006	**−0.54**
	Solid Water 3	1.00	7.45	1.002 ± 0.002	–	–	–	0.996	−0.006	**−0.60**
	Solid Water 4	1.00	7.45	1.002 ± 0.002	–	–	–	0.996	−0.005	**−0.53**
	True Water 1	1.00	7.42	1.000 ± 0.000	–	–	–	1.004	0.004	**0.35**
	True Water 2	1.00	7.42	1.000 ± 0.000	–	–	–	1.003	0.003	**0.27**
Advanced Electron Density Head	Adipose	0.94	6.61	0.960 ± 0.002	0.955	−0.005	**−0.51**	0.956	−0.004	**−0.46**
	Brain	1.02	7.63	1.028 ± 0.002	1.028	0.000	**0.00**	1.029	0.001	**0.11**
	Breast	0.97	6.92	0.981 ± 0.002	0.975	−0.006	**−0.60**	0.976	−0.005	**−0.49**
	CaCO3 30%	1.27	10.78	1.261 ± 0.002	1.255	−0.005	**−0.42**	1.258	−0.002	**−0.18**
	CaCO3 50%	1.46	12.40	1.451 ± 0.002	1.429	−0.022	**−1.50**	1.432	−0.018	**−1.26**
	Cortical bone	1.78	13.63	1.699 ± 0.002	1.720	0.021	**1.27**	1.722	0.023	**1.33**
	Solid Water 1	1.00	7.45	1.002 ± 0.002	1.000	−0.002	**−0.16**	0.998	−0.003	**−0.33**
	Solid Water 2	1.00	7.45	1.002 ± 0.002	0.997	−0.005	**−0.48**	0.998	−0.004	**−0.38**
	Solid Water 3	1.00	7.45	1.002 ± 0.002	0.999	−0.003	**−0.27**	0.999	−0.003	**−0.30**
	True Water 1	1.00	7.42	1.000 ± 0.000	1.004	0.004	**0.36**	1.004	0.004	**0.37**

Abbreviations: SFOV, Scanning Field of View.

**FIGURE 2:**
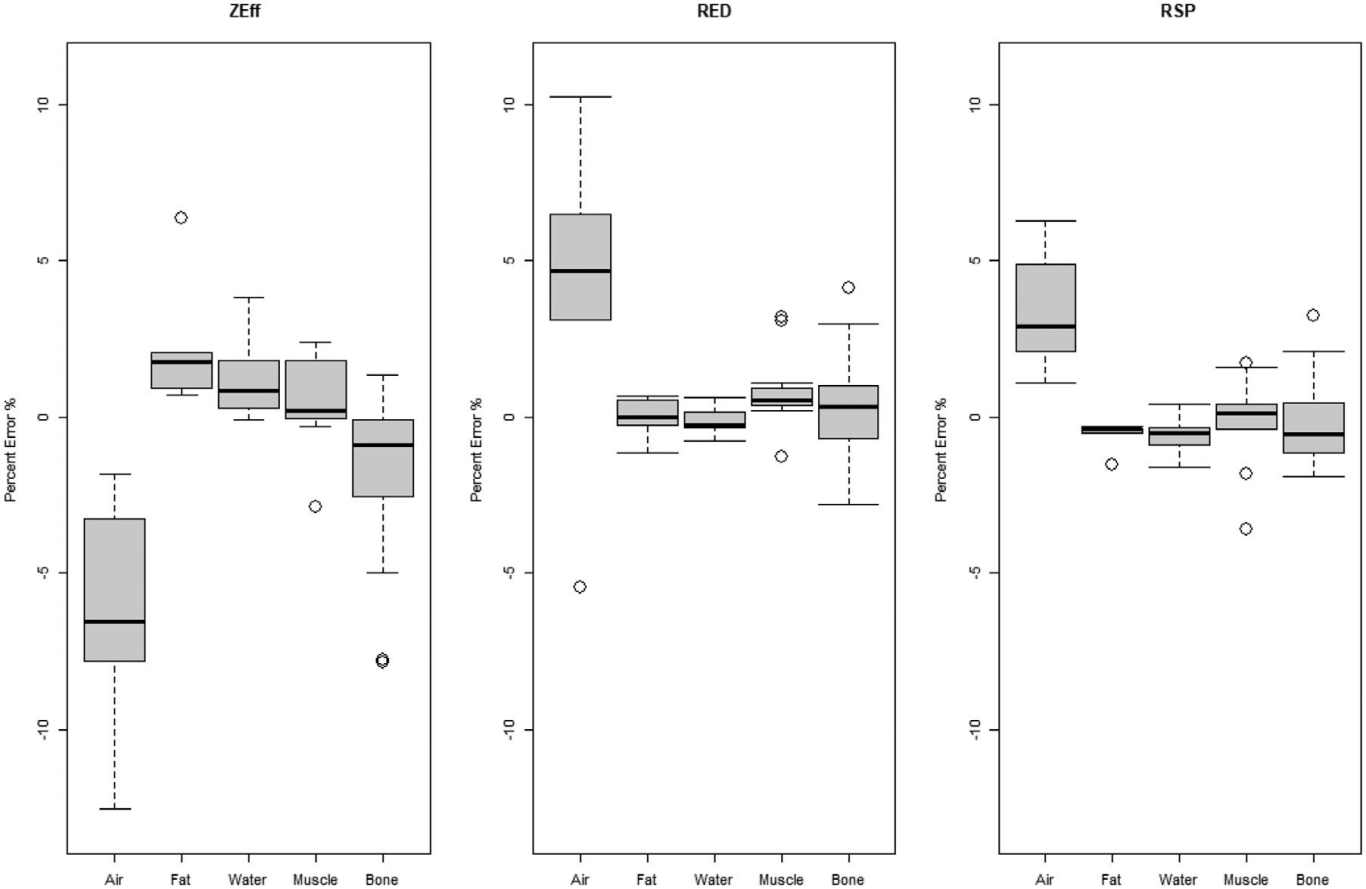
Box and whisker plots displaying the percent error between measured DECT values compared to the reference $Z_{\mathrm{eff}}$, RED, RSP for air/lung, fat, water, muscle and bone for SFOV body filter. The horizontal line in each box indicates the median value that separates the upper and lower quartiles. The whiskers represent the minimum and maximum value of the interquartile range. Dots represent outliers. DECT, dual energy computed tomography; RED, relative electron density; RSP, relative stopping power; SECT, single‐energy computed tomography; SFOV, Scanning Field of View.

Figure 2 displays the spread of the percent error categorized by tissue type for $Z_{\mathrm{eff}}$, RED, and RSP for all of the inserts scanned with the Body SFOV. The overall percent error and the standard deviation for $Z_{\mathrm{eff}}$, RED, and RSP were found to be −0.52% ± 3.17%, 0.58% ± 2.19%, and 0.02% ± 1.63%, respectively. The largest percent error seen across all values for $Z_{\mathrm{eff}}$, RED, and RSP was in the low‐density inserts with a RSP MAPE of 3.46% for air/lung. Table 2 displays the MAPE of $Z_{\mathrm{eff}}$, RED, and RSP for air/lung, fat, water, muscle, bone using the Body SFOV. The overall RSP MAPE for all tissue‐mimicking plugs is 1.13 ± 1.17%.

**TABLE 2:** Mean absolute percent error of tissue mimicking inserts body bowtie filter.

ROI	RED	MAPE $Z_{\mathrm{eff}}$	MAPE RED	MAPE RSP
Air/Lung	0.28‐0.44	6.39	6.00	3.46
Fat	0.93‐0.97	2.26	0.46	0.60
Water	0.99‐1.00	1.17	0.31	0.68
Muscle	1.02‐1.08	0.92	1.17	0.99
Bone	1.09‐1.78	2.10	1.15	1.12
	Overall MAPE	2.02 ± 2.48	1.29 ± 1.86	1.13 ± 1.17

Abbreviations: MAPE, mean absolute percent error; RED, relative electron density; RSP, relative stopping power; $Z_{\mathrm{eff}}$, effective atomic number.

### 3.2 Change in SFOV

**FIGURE 3:**
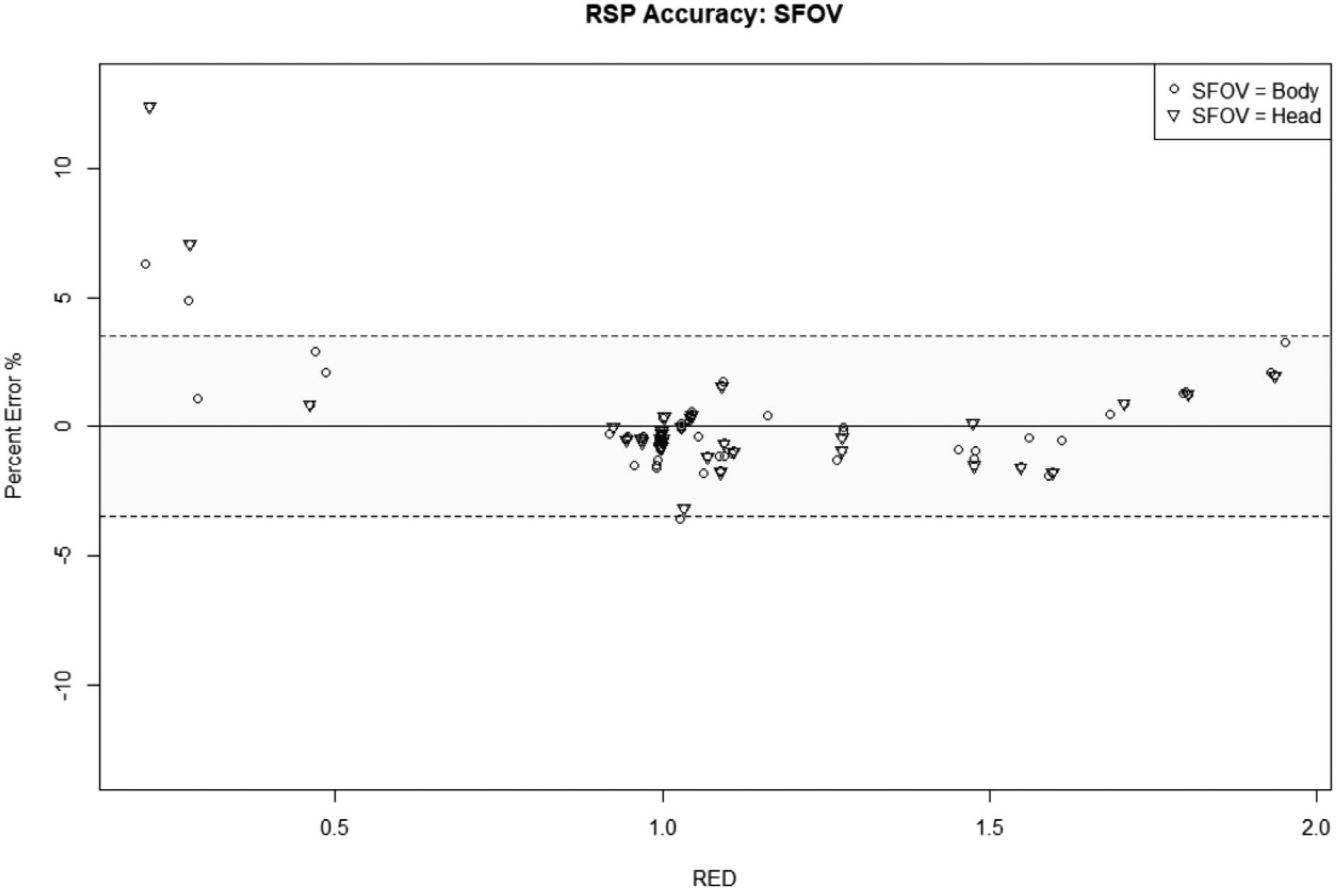
RSP accuracy shown for all of the inserts scanned using either a body or head SFOV plotted from low to high RED. The shaded area represents the typical uncertainty of ±3.5% used with SECT planning. RED, relative electron density; RSP, relative stopping power; SECT, single‐energy computed tomography; SFOV, Scanning Field of View.

The average residual error of the plugs scanned using Head SFOV is −0.002 ± 0.015. The distribution of differences for identical plugs, but acquired using Body SFOV was compared using the paired Student's *t*‐test. There was no significant difference in accuracy scanning with a Body SFOV versus a Head SFOV (*p* = 0.61). The average residual error of the plugs scanned with Head SFOV scanned with the Body SFOV is −0.003 ± 0.016. Similar to the results from the Body SFOV, the air/lung inserts had the largest percent error in RSP with the largest percent error seen in the sinus and LN‐300 inserts at 12.4% and 7.07%, respectively. Figure 3 displays the percent error across all of the plugs according to their specified RED for both the Body and the Head SFOV. Most points fell within ±3.5% with exception to low density air/lung inserts for both Body and Head SFOV.

**FIGURE 4:**
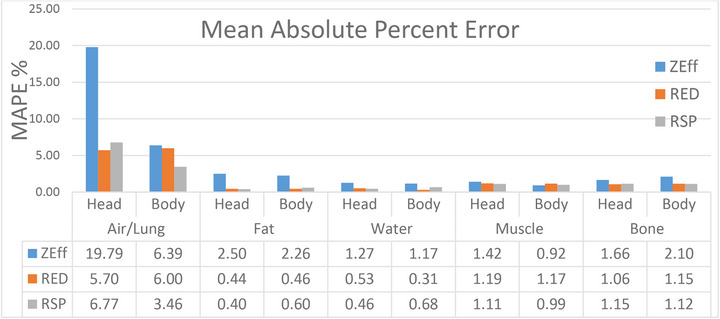
$Z_{\mathrm{eff}}$, RED, and RSP MAPE across all tissue categories and bow tie filter used. For all categories, the air/lung had the highest error. All other tissues had a MAPE within 2.5% for $Z_{\mathrm{eff}}$ and within 1.2% for RED and RSP. MAPE, mean absolute percent error; RED, relative electron density; RSP, relative stopping power; $Z_{\mathrm{eff}}$, effective atomic number.

Figure 4 summarizes the MAPE for all $Z_{\mathrm{eff}}$, RED, and RSP at each SFOV for each density category. Air/lung exhibited the largest MAPE for each SFOV for all $Z_{\mathrm{eff}}$, RED, and RSP. Excluding air/lung, RSP MAPE is less than 1.2%.

## 4 DISCUSSION

In this study, the accuracy of a fast kV switching DECT in determining $Z_{\mathrm{eff}}$, RED, and RSP was evaluated across several tissue‐mimicking inserts in phantoms of various shapes and sizes. This was done using the same scanning thickness and the highest available mA setting with a change in either Body or Head SFOV. Although these estimates were made on tissue‐equivalent phantoms, the difference between homogeneous tissue‐equivalent material and actual human tissue composition may vary.^1^, ^24^

In a study by Landry et al., the $Z_{\mathrm{eff}}$ accuracy was up to 10% using a DECT scanner.^7^ Tatsugami et al. analyzed the accuracy in $Z_{\mathrm{eff}}$ and RED on a dual‐rotation CT scanner and found the $Z_{\mathrm{eff}}$ accuracy to be approximately 8% and RED accuracy up to 5%.^14^ Ohira et al. analyzed the accuracy of a first‐generation dual layer CT for $Z_{\mathrm{eff}}$, RED, and RSP and found the largest error in $Z_{\mathrm{eff}}$, which ranged from −6.4 ±8%.^15^ This is similar to the results in Figure 4, where the largest $Z_{\mathrm{eff}}$ MAPE was in air/lung for both Head and Body SFOV at 19.79% and 6.39%, respectively. The largest MAPE in RED was 5.7% in Head and 6% in Body SFOV. The largest RSP errors were found in low density air/lung with a MAPE of 3.46% for Body and 6.77% in Head SFOV.

One reason for large error in lung tissue is due to its low density and its susceptibility to noise. An increase in noise increases the variability in the calculated $Z_{\mathrm{eff}}$ and RED CT number value that is computed using the low and high energy spectrum of the DECT. Therefore, any noise that exists in either energy scan of the DECT will propagate into the overall accuracy of the final derived RSP value. Scans were intentionally set with the highest mA available in order to minimize the impact of noise despite the use of phantoms of various sizes. In a clinical setting, optimizing mA is essential for establishing protocols for size‐specific anatomical regions. This involves selecting mA settings that utilize the lowest dose that is reasonably achievable without compromising image quality. Many modern scanners use protocols that utilize a variable mA that adjusts as patient attenuation changes to maintain a consistent noise level for all slices. A decrease in mA for a set of scanning parameters will increase noise and therefore increase the variability and potentially the accuracy of the calculated RSP. Because the goal of the study is to evaluate the accuracy of the fast kV switching DECT in calculating RSP in different‐sized phantoms, the use of the highest mA setting available sets the upper limit of expected accuracy by reducing noise as much as mechanically possible. The impact of noise using a variety of low‐dose clinical protocols on RSP accuracy should be further investigated and evaluated prior to clinical use and falls outside the scope of the present work. The noise was measured as the standard deviation of a 2.8 cm circle at the center of the phantom. For the Advanced Electron Density Head phantom, because the center of the phantom was a solid water insert, a smaller 1 cm circle centered inside the insert and away from the edge was used. Table 3 summarizes the impact of the various phantom sizes and CTDI_vol_ on noise. For the same phantom size, the increase in CTDI_vol_ resulted in a decrease in noise. The results of the average and standard deviation of the residual error in 3.2 showed a slight improvement in the higher‐dose scan compared to the lower dose. For scans with the same CTDI_vol_, noise was higher in the larger phantom due to the effects of beam hardening and attenuation through more material. The largest σ_RSP_ of 0.018 was found in both the largest phantom and the scan with the lowest dose. Table 1 shows that the residual error of the air/lung inserts using the Body SFOV for the Advanced Electron Density and Model 467 is within the observed noise. However, the sinus insert in the George phantom had a residual error higher than the measured noise. This can be attributed to its proximity to high‐density structures.

**TABLE 3:** Noise (*σ*) as a function of phantom diameter (cm) and dose (mGy).

Phantom	Diameter (cm)	CTDI_vol_ (mGy)	*σ*_Zeff_	*σ*_RED_	*σ*_RSP_
Advanced Electron Density Body	34.6	**18.85**	0.636	0.023	0.018
Advanced Electron Density Head	**20**	**18.85**	0.153	0.008	0.008
Advanced Electron Density Head	**20**	82.77	0.087	0.004	0.004
Model 467	**33**	14.55	0.496	0.021	0.018
Model 467	**33**	82.77	0.223	0.011	0.010
George Body	27	**18.85**	0.382	0.010	0.007
George Head	**18**	**18.85**	0.240	0.007	0.006
George Head	**18**	82.77	0.028	0.003	0.003

Abbreviations: RED, relative electron density; RSP, relative stopping power; $Z_{\mathrm{eff}}$, effective atomic number.

Another contribution that impacts final CT number is scatter artifact from nearby high‐density structures. In the George phantom, the low‐density sinus plug showed the highest percent error of 6.3%. This plug was located next to dental dentin, cortical bone, trabecular bone, and dental enamel. As observed in Figure 2, air/lung displayed the largest spread, which could be contributed to the surrounding scatter conditions. For each lung and sinus insert evaluated, beam hardening effects play a role in the final calculation and a different location or absence of these high‐density materials may improve the RSP accuracy. This can be observed in the Model 467 phantom. The LN‐300 plug was located closer to cortical bone than in the Advanced Electron Density phantom configuration. The percent error in the RSP was 2.75%, while in the Advanced Electron Density phantom configuration LN‐300 was 1.11%. In another example, the RSP residual error of the brain insert from Model 467 was greater than 2 SD compared to the brain inserts in the other phantoms. Those brain inserts were at least 3 cm away from nearby high‐density inserts, whereas in the Model 467 the brain insert was only 1.4 cm away from cortical bone.

The RSP residual error of enamel was also shown to have a statistically significant deviation with more than 2 SD from the known RSP. The enamel insert had the highest RED of 1.875 and the highest $Z_{\mathrm{eff}}$ at 17.2. The accuracy of mapping $Z_{\mathrm{eff}}$ and RED results from the information from low and high kVp of the dual energy CT. Not only do beam hardening effects increase with high‐density material, but also with a higher $Z_{\mathrm{eff}}$, photoelectric effect increases, which reduces the accuracy of DECT material decomposition.^5^, ^25^ More data is needed to evaluate the accuracy at even higher Z materials.

One advantage of the fast kV switching DECT is the ability to acquire a large FOV.^6^, ^23^ In radiation therapy treatment planning, the entire patient and all devices in the beam path must be included in the field of view during a treatment planning simulation CT. For this reason, the SFOV Large Body would commonly be used. The Head SFOV would only be incorporated clinically if the area being treated, including devices, could fit within the field of view. Head bowtie filters are optimized for a smaller field of view. An advantage of using the Head SFOV is with increased spatial resolution available to the smaller FOV. However, a smaller voxel size and any mis‐centering of the head will lead to an increase in noise. In this study, all phantoms were centered in the bore to ensure optimal use of the bowtie filter. Bowtie filters are vital for improving uniformity in an image by compensating for the size and round nature of a patient. The shape of the bowtie filter results in the loss of dose at the periphery with the assumption that the patient is centered in the bore.^26^ It is important to consider the size and shape of what is being scanned and the potential effects of the bowtie filter. The Model 467 phantom did not have a separate smaller head insert like the George and Advanced Electron Density phantoms. Its 33 cm diameter was slightly larger than the SFOV of the 32 cm Head bowtie filter. Lung inserts were located on the outer periphery of the phantom and were included in both the Body and Head SFOV scan evaluations. The RSP percent error of the LN‐300 in Model 467 was 2.75% and when it was rescanned with the Head SFOV, the percent error increased to 7.07%. With a Body bowtie filter, more low‐energy photons are attenuated to harden the beam in order to improve dose and fluence uniformity on a large body. With the Head SFOV, less low energy photons are attenuated in order to improve contrast in a head. Therefore, the Head SFOV is more sensitive to beam hardening. The sinus plug, when scanned with the Body SFOV, displayed the highest error at 6.3% due to its proximity to high‐density objects as discussed. When scanned with the Head SFOV, the RSP percent error increased to 12.3% as a result of the difference in beam hardening correction that each filter provides. Each institution must evaluate the use of a Head bowtie filter and the effects on RSP accuracy for their patient setup.

The overall RSP percent accuracy across all inserts was 0.02% ± 1.63 consistent with previously investigated DECTs, which ultimately concluded reasonable RSP accuracy.^7^, ^8^, ^9^, ^10^, ^11^, ^12^, ^13^, ^14^, ^15^, ^16^ For a dual‐source scanner and dual‐rotation scanner, an additional registration step is required to process the low‐ and high‐energy spectra.^6^ Any patient motion that may occur between the time delay during the acquisition of the two energy spectra will result in artifacts, which cause significant errors in the final RSP calculation.^5^, ^6^ For a single‐source acquisition, such as the fast kV switching DECT or dual layer CT, misregistration artifacts are avoided.^6^ However, the dual layer CT is limited to a more narrow spectral separation based on the detector design, which lowers SNR.^25^ The fast kV switching DECT is advantageous for proton therapy treatment planning as it results in more distinct energy separation by generating the high and low energies separately.^5^, ^6^ The latest technology in CT, which offers the advantage of higher spectral separation while also mitigating registration issues by using a single source, is photon counting detector (PCD) technology. PCD can distinguish the energy of each photon, which allows for Multi Energy CT.^25^, ^27^ PCD‐CT offers benefits over DECT, such as higher spatial resolution, improved noise reduction, and energy weighting, which improves contrast.^25^, ^27^ Preliminary studies show the potential of PCD‐CT to accurately determine $Z_{\mathrm{eff}}$ and RED.^25^, ^27^, ^28^ In the same manner as DECT, by applying Equations 1 and 2 above, PCD‐CT also has the potential to accurately determine RSP. In a study by Hu, on a first‐generation PCD‐CT, RSP was calculated to have an error of 1.27% with the largest error in lung at −3.9%.^28^

## 5 CONCLUSIONS

The fast kV switching dual energy CT was shown to be accurate in calculating $Z_{\mathrm{eff}}$, RED, and RSP. Scattering conditions impacted the accuracy of low‐density material. However, the air/tissue inserts were shown to be non‐inferior to single‐energy CT, while fat, water, muscle, and bone was an improvement to commonly used uncertainties seen in single‐energy CT. An evaluation of scanning with a Body or Head SFOV was found to show no significant difference (*p* > 0.6). However, it is important for each institution to understand its accuracy on RSP and clinical use for their DECT scanner type.

## CONFLICT OF INTEREST STATEMENT

The authors declare no conflicts of interest.

## References

[1] Yang M , Zhu XR , Park PC , et al. Comprehensive analysis of proton range uncertainties related to patient stopping‐power‐ratio estimation using the stoichiometric calibration. Phys Med Biol. 2012;57(13):4095‐4115. doi:10.1088/0031-9155/57/13/4095 22678123 PMC3396587

[2] Moyers MF , Sardesai M , Sun S , Miller DW . Ion stopping powers and CT numbers. Med Dosim. 2010;35(3):179‐194. doi:10.1016/j.meddos.2009.05.004 19931030

[3] Paganetti H . Range uncertainties in proton therapy and the role of Monte Carlo simulations. Phys Med Biol. 2012;57(11):R99‐R117. doi:10.1088/0031-9155/57/11/R99 22571913 PMC3374500

[4] Schneider U , Pedroni E , Lomax A . The calibration of CT Hounsfield units for radiotherapy treatment planning. Phys Med Biol. 1996;41(1):111‐124. doi:10.1088/0031-9155/41/1/009 8685250

[5] Johnson TRC . Dual‐energy CT: general principles. Am J Roentgenol. 2012;199(5 suppl):S3‐S8. doi:10.2214/AJR.12.9116 23097165

[6] Teo K . Dual Energy Computed Tomography for Proton Dose Calculation [Internet]. Published 2020. Accessed September 17. AAPM; 2023. http://amos3.aapm.org/abstracts/pdf/155‐53961‐1531640‐157689.pdf

[7] Landry G , Seco J , Gaudreault M , Verhaegen F . Deriving effective atomic numbers from DECT based on a parameterization of the ratio of high and low linear attenuation coefficients. Phys Med Biol. 2013;58(19):6851‐6866. doi:10.1088/0031-9155/58/19/6851 24025623

[8] Bär E , Lalonde A , Royle G , Lu HM , Bouchard H . The potential of dual‐energy CT to reduce proton beam range uncertainties. Med Phys. 2017;44(6):2332‐2344. doi:10.1002/mp.12215 28295434

[9] Lalonde A , Bouchard H . A general method to derive tissue parameters for Monte Carlo dose calculation with multi‐energy CT. Phys Med Biol. 2016;61(22):8044‐8069. doi:10.1088/0031-9155/61/22/8044 27779137

[10] Bazalova M , Carrier JF , Beaulieu L , Verhaegen F . Dual‐energy CT‐based material extraction for tissue segmentation in Monte Carlo dose calculations. Phys Med Biol. 2008;53(9):2439‐2456. doi:10.1088/0031-9155/53/9/015 18421124

[11] Hünemohr N , Krauss B , Tremmel C , Ackermann B , Jäkel O , Greilich S . Experimental verification of ion stopping power prediction from dual energy CT data in tissue surrogates. Phys Med Biol. 2014;59(1):83‐96. doi:10.1088/0031-9155/59/1/83 24334601

[12] Xie Y , Ainsley C , Yin L , et al. Ex vivo validation of a stoichiometric dual energy CT proton stopping power ratio calibration. Phys Med Biol. 2018;63(5):055016. doi:10.1088/1361-6560/aaae91 29513647

[13] Bourque AE , Carrier JF , Bouchard H . A stoichiometric calibration method for dual energy computed tomography. Phys Med Biol. 2014;59(8):2059‐2088. doi:10.1088/0031-9155/59/8/2059 24694786

[14] Tatsugami F , Higaki T , Kiguchi M , et al. Measurement of electron density and effective atomic number by dual‐energy scan using a 320‐detector computed tomography scanner with raw data‐based analysis: a phantom study. J Comput Assist Tomogr. 2014;38(6):824‐827. doi:10.1097/RCT.0000000000000129 24983439

[15] Ohira S , Washio H , Yagi M , et al. Estimation of electron density, effective atomic number and stopping power ratio using dual‐layer computed tomography for radiotherapy treatment planning. Phys Med. 2018;56:34‐40. doi:10.1016/j.ejmp.2018.11.008 30527087

[16] Li B , Lee HC , Duan X , et al. Comprehensive analysis of proton range uncertainties related to stopping‐power‐ratio estimation using dual‐energy CT imaging. Phys Med Biol. 2017;62(17):7056‐7074. doi:10.1088/1361-6560/aa7dc9 28678019 PMC5736379

[17] Wang H , Qu Y , Li Y , Deak P , Pankuch M . Validation of derived relative stopping power using fast switch KV dual energy CT for proton planning. NY International Journal of Particle Therapy. 2024;15:100646. doi:10.1016/j.ijpt.2024.100646. Abstract presented at: PTCOG‐NA. PMC12688056 41377697

[18] Gammex . Tissue Characterization Phantom Gammex Model 467 User's Guide. Gammex; 2004

[19] Gammex . User's Guide, Advanced Electron Density Phantom. Gammex Inc.; 2017.

[20] Dedes G , Drosten H , Götz S , et al. Comparative accuracy and resolution assessment of two prototype proton computed tomography scanners. Med Phys. 2022;49(7):4671‐4681. doi:10.1002/mp.15657 35396739

[21] DeJongh DF , DeJongh EA , Rykalin V , et al. A comparison of proton stopping power measured with proton CT and x‐ray CT in fresh postmortem porcine structures. Med Phys. 2021;48(12):7998‐8009. doi:10.1002/mp.15334 34739140 PMC8678357

[22] Pasquier H , Liu EH . Supercharge Spectral CT with TrueFidelity for Gemstone Spectral Imaging (GSI) [White paper]. Published August 2023. Accessed July 9. GE HealthCare; 2025. https://events.gehealthcare.com/wp‐content/uploads/2023/08/TF‐GSI‐Evidence‐White‐Paper‐Final‐JB23533XX_22Aug2023.pdf

[23] Thibault JB , Nett B , Tang J , Liu E . TrueFidelity DL for Spectral Imaging [White paper]. Published November 2023. Accessed July 9. GE HealthCare; 2025. https://www.gehealthcare.com.au/‐/jssmedia/gehc/us/files/products/computed‐tomography/revolution‐family/revolution‐apex/truefidelity‐for‐gsi‐whitepaper_digital_jb19879xx_v12_13nov2023.pdf

[24] Yang M , Virshup G , Clayton J , Zhu XR , Mohan R , Dong L . Theoretical variance analysis of single‐ and dual‐energy computed tomography methods for calculating proton stopping power ratios of biological tissues. Phys Med Biol. 2010;55(5):1343‐1362. doi:10.1088/0031‐9155/55/5/006 20145291 10.1088/0031-9155/55/5/006

[25] Richtsmeier D , Rodesch PA , Iniewski K , Bazalova‐Carter M . Material decomposition with a prototype photon‐counting detector CT system: expanding a stoichiometric dual‐energy CT method via energy bin optimization and K‐edge imaging. Phys Med Biol. 2024;69(5):055001. doi:10.1088/1361‐6560/ad25c8 10.1088/1361-6560/ad25c8 38306974

[26] Boone JM . Method for evaluating bow tie filter angle‐dependent attenuation in CT: theory and simulation results. Med Phys. 2010;37(1):40‐48. doi:10.1118/1.3264616 20175464 10.1118/1.3264616 PMC2801732

[27] Garnett R . A comprehensive review of dual‐energy and multi‐spectral computed tomography. Clin Imaging. 2020;67:160‐169. doi:10.1016/j.clinimag.2020.07.030 32795784 10.1016/j.clinimag.2020.07.030

[28] Hu G , Niepel K , Risch F , et al. Assessment of quantitative information for radiation therapy at a first‐generation clinical photon‐counting computed tomography scanner. Front Oncol. 2022;12:970299. doi:10.3389/fonc.2022.970299 36185297 10.3389/fonc.2022.970299 PMC9515409

